# Training peripheral vision to read: Using stimulus exposure and identity priming

**DOI:** 10.3389/fnins.2022.916447

**Published:** 2022-08-24

**Authors:** Deyue Yu

**Affiliations:** College of Optometry, The Ohio State University, Columbus, OH, United States

**Keywords:** peripheral vision, reading speed, perceptual learning, crowding, reading rehabilitation

## Abstract

Reading in the periphery can be improved with perceptual learning. A conventional training paradigm involves repeated practice on a character-based task (e.g., recognizing random letters/words). While the training is effective, the hours of strenuous effort required from the trainees makes it difficult to implement the training in low-vision patients. Here, we developed a training paradigm utilizing stimulus exposure and identity priming to minimize training effort and improve training accessibility while maintaining the active engagement of observers through a stimulus visibility task. Twenty-one normally sighted young adults were randomly assigned to three groups: a control group, a with-repetition training group, and a without-repetition training group. All observers received a pre-test and a post-test scheduled 1 week apart. Each test consisted of measurements of reading speed, visual-span profile, the spatial extent of crowding, and isolated-letter profiles at 10° eccentricity in the lower visual field. Training consists of five daily sessions (a total of 7,150 trials) of viewing trigram stimuli (strings of three letters) with identity priming (prior knowledge of target letter identity). The with-repetition group was given the option to replay each stimulus (averaged 0.4 times). In comparison to the control group, both training groups showed significant improvements in all four performance measures. Stimulus replay did not yield a measurable benefit on learning. Learning transferred to various untrained tasks and conditions, such as the reading task and untrained letter size. Reduction in crowding was the main basis of the training-related improvement in reading. We also found that the learning can be partially retained for a minimum of 3 months and that complete retention is attainable with additional monthly training. Our findings suggest that conventional training task that requires recognizing random letters or words is dispensable for improving peripheral reading. Utilizing stimulus exposure and identity priming accompanied by a stimulus visibility task, our novel training procedure offers effective intervention, simple implementation, capability for remote and self-administration, and an easy translation into low-vision reading rehabilitation.

## Introduction

While visual functions are shaped by adverse experiences such as eye disease, the functional properties of the visual system can also be altered *via* designed experience. For people with central vision loss who rely exclusively on peripheral vision, reading is slow and difficult. A number of training methods have been developed to enhance reading performance in these patients, including assistive device training (Nilsson, 1990; Cheong et al., 2005), oculomotor training (Seiple et al., 2005; Rosengarth et al., 2013), and preferred retinal locus training (Nilsson et al., 1998, 2003). Recently, perceptual learning, a long-term enhancement in perception and behavior arising from repeated practice or sensory experience (Gibson, 1963; Karni and Sagi, 1991; Sagi, 2011), has been introduced as a potential alternative method for low-vision reading rehabilitation. The positive results observed in many studies have attested to the utility of perceptual learning intervention in improving reading performance in the normal periphery (e.g., Yu et al., 2010a; Subramanian et al., 2014) and in people with central vision loss (e.g., Chung, 2011; Nguyen et al., 2011; Tarita-Nistor et al., 2014; Maniglia et al., 2020), with more showing the potential and effectiveness of perceptual learning in low-vision rehabilitation (e.g., Maniglia et al., 2011, 2016; Plank et al., 2014).

Outcome effectiveness is the predominant objective when designing a training paradigm, whereas ease of implementation is the major hurdle for successful translation of the training into effective low-vision rehabilitation intervention. A conventional perceptual learning paradigm for improving reading in peripheral vision involves repeated practice on a character-based task, such as recognizing letters in trigrams (random strings of three letters) (e.g., Yu et al., 2010b,^2017^). Following an intensive period of training (e.g., several hours over multiple days), observers demonstrate significant improvement in reading speed using their peripheral vision. While the training is effective, the hours of strenuous effort required from the observers makes it difficult to implement the training in low-vision patients. Furthermore, perceptual learning-based training would require multiple, regular clinic visits which can be particularly challenging for these patients. To improve training accessibility and ensure good compliance, it is necessary to move such intervention to a home-based setting and develop a protocol that offers self-administration with greater ease. This need raises an important question of whether and how we can minimize training effort and achieve self-administration. While a few home-based training programs have been developed for low-vision reading (e.g., Goodrich et al., 2004; Reeves et al., 2004; Coco-Martín et al., 2013), none were targeting to improve reading-related sensory factors using perceptual learning. Here, we develop a training procedure utilizing stimulus exposure and identity priming (prior knowledge of stimulus identity) accompanied by a stimulus visibility task. Our results show that this new training procedure offers a more economic approach to achieve our training goal. It can provide significant, transferrable, and lasting improvements while giving the trainee complete control over the course of training and requiring minimal effort and less training time from the trainee.

Perceptual learning involves a broad network of brain regions, and its behavioral and neurological outcomes can differ significantly depending on the details of the training procedure and the characteristics of the individual trainee (see review by Maniglia and Seitz, 2018). Amitay et al. (2006) proposed that optimal perceptual learning requires the inclusion of three processes: stimulus exposure, attention to relevant stimulus dimension (e.g., for reading, it can be the identity of letters), and general arousal. Here, we design the training protocol with these considerations in mind.

As in the previous training studies (e.g., Yu et al., 2010b,^2017^), we adopt trigrams as the training stimuli. To minimize training effort, we keep only the stimulus exposure component and remove the associated recognition task. Despite not requiring recognizing random letters or words, our perceptual learning paradigm involves components ensuring observers’ attention being directed to reading relevant stimulus dimension (through goal setting – learning to identify crowded letters), observers’ active engagement (through a stimulus visibility task), and reinforcement signals (through internal feedback).

Top-down control can assist both conscious visual perception and visual perceptual learning (Ahissar and Hochstein, 2004). When designing the training paradigm, we utilize the top-down guidance mechanism to maximize the benefit of stimulus exposure. Specifically, we adopt identity priming (prior knowledge of stimulus identity). With identity priming, observers not only have knowledge of the forthcoming stimulus but also generate internal feedback on their visual performance (i.e., the information regarding their ability to discern the target letter) following each stimulus presentation. Feedback has been considered as an important factor in perceptual learning. Although learning can occur without feedback (e.g., Yu et al., 2010b), providing feedback can facilitate learning (increase the rate, extent, and stabilization of learning) (Ball and Sekuler, 1987; Herzog and Fahle, 1997, 1998, 1999; Dobres and Watanabe, 2012) or even directly induce learning (Choi and Watanabe, 2012). In our training paradigm, identity priming allows the feedback component to be fulfilled. Essentially, our observers undertake self-supervised learning. With the proposed design, we expect that our training will provide robust learning against deterioration and interference, and offer a stable improvement over the long term.

In peripheral vision, shrinkage of the visual span (the reduced number of letters that can be recognized reliably within a single fixation) is associated with slower reading speed (Legge et al., 2001). It indicates that expanding visual span may lead to faster peripheral reading speed. Indeed, several studies have confirmed this hypothesis. Enlarging the visual span through training on a letter recognition task in the periphery is consistently accompanied by an improvement in reading speed (e.g., Yu et al., 2010b; He et al., 2013). The size of the visual span is determined by three sensory factors: crowding, mislocations (errors in the spatial order of letters within a stimulus), and decreasing resolution with eccentricity (Yu et al., 2014). Crowding describes the adverse interference effect of adjacent letters on target letter recognition. Among the three sensory determinants, reduction of crowding accounts for the majority of the training-related improvements in the size of the visual span and reading speed (He et al., 2013). If this observation is not contingent on the involvement of recognizing random letters or words during training, we should expect similar findings in the proposed training.

As shown in previous reading training studies, learning can transfer to untrained tasks and conditions (e.g., Yu et al., 2010a,b; Tarita-Nistor et al., 2014; Maniglia et al., 2020), and can be retained for at least 3 months (e.g., Chung et al., 2004). The generalization and retention of learning confirmed the suitability of the training in a real-world application. Although we have learned much from previous research, it is necessary to evaluate the generalization and retention of learning for the proposed training. For generalization, we are particularly interested in the transfer of learning to tasks (e.g., reading words) performed at smaller print sizes because the print sizes used in daily reading materials are mostly small (Legge and Bigelow, 2011). The enhancement in the ability to read smaller fonts has been associated with the improved score of self-reported quality of life in patients with central vision loss following a reading rehabilitation program (Coco-Martín et al., 2013).

The present study investigates whether the proposed new training method (utilizing stimulus exposure and identity priming) offers effective intervention in the normal periphery of young adults. Specifically, through a battery of tasks, we evaluate how reading speed and the reading-related sensory factors change following our novel training procedure. We also examine several subsidiary questions: (1) whether the improvements generalize to untrained stimuli, tasks, and print size, (2) whether replaying training stimuli yield any measurable benefit to learning, (3) how learning distributes among the three sensory factors, (4) whether learning can be retained for a long period of time, and (5) whether additional monthly training facilitates the retention of learning. The findings of this study are of practical importance for developing a useful form of reading rehabilitation in patients with central vision loss.

## Methods

### Observers

Twenty-one native English speakers (aged 18–27 years; 11 women and 10 men) with normal or corrected-to-normal vision participated in the study. The sample size (seven observers per group) was pre-determined based on standard statistical power analysis and existing studies on similar topics and observer populations (e.g., Chung et al., 2004; Yu et al., 2010b,^2017^). None of the observers had prior experience in the tasks used in the current study or participated in experiments involving testing peripheral vision. Monetary compensation was provided to observers based on the time of participation. Prior to the experiment, all observers granted written consent after the procedures of the experiment were explained. The experimental protocol conformed to the tenets of the Declaration of Helsinki and was approved by the Institutional Review Board at the Ohio State University.

### Apparatus and stimuli

Stimuli were created on a Macintosh computer using custom functions written in MATLAB R2010a using the Psychtoolbox 3 (Brainard and Vision, 1997; Pelli, 1997; Kleiner et al., 2007). All stimuli were black text on a white background, and were presented on a 21″ ViewSonic Graphics Series G225f CRT monitor (resolution: 1,280 × 1,024; refresh rate: 85 Hz). Letters were rendered in lowercase Courier font (a fixed-width, serif font). Testing was performed binocularly in a dark room. A chin and headrest were used to maintain a constant viewing distance of 40 cm.

### Experimental design

**FIGURE 1:**
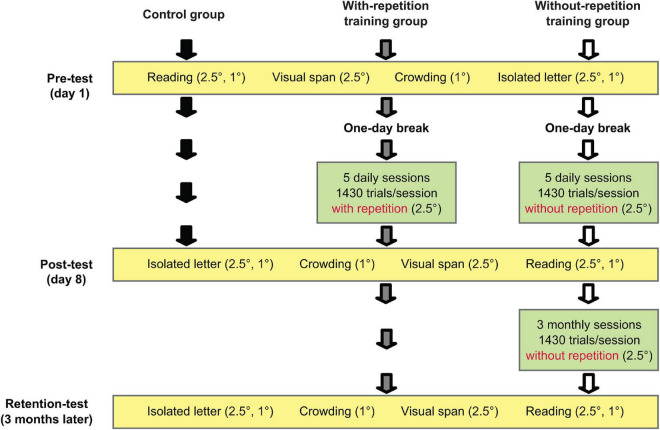
Experiment design.

A schematic of the experimental design is shown in Figure 1. Our observers were randomly assigned to three groups with seven observers in each group: a control group, a with-repetition training group, and a without-repetition training group. All observers received a pre-test and a post-test scheduled 1 week apart. The pre-test consisted of measurements of reading speed, visual-span profile, the spatial extent of crowding, and isolated-letter profile (in the order given). All tests were performed at 10° eccentricity in the lower visual field. The two training groups received daily training sessions at 10° in the lower field on 5 consecutive days, starting on the 2nd day after the pre-test. The post-test was performed on the day after the 5th training session. The post-test was identical to the pre-test except that the measurements were obtained in the reversed order. The control group participated only in the pre- and post-tests and had no intervening training. We included the control group because it is possible that the pre-test experience on its own may provide some improvement in performance.

The tasks used to measure reading speed, visual-span profile, the spatial extent of crowding, and isolated-letter profile are Rapid Serial Visual Presentation (RSVP), trigram, crowded letter recognition, and isolated letter recognition, respectively. Each task was proceeded by several practice trials to accustom the observers to the task. During the experiment, except for the training sessions, observers’ eye positions were monitored by an experimenter who can reliably detect eye movements of 1.5 or larger from about 40 cm away. The testing trial was canceled and replaced upon the detection of any deviation away from the fixation target.

A print size of 2.5° (defined as lowercase x-height), larger than the critical print size (CPS) for reading at 10° eccentricity (Chung et al., 1998), was used in the study except when measuring the spatial extent of crowding (1° print size). We also measured reading speed and isolated-letter profile at 1° print size to evaluate the possible transfer of learning from a trained (2.5°) to an untrained print size (1°). The testing order of the print sizes was balanced across the observers.

To investigate whether the training-related improvements could be retained over an extended period of time, all observers in the with-repetition training group and five observers in the without-repetition training group returned about 3 months after the post-test for a retention-test that was identical to the post-test. A previous reading training study (Chung et al., 2004) tracked a subgroup of observers for performance changes at 1 week, 1 month, and 3 months after the post-test. While they found sizable retention of the learning in trained observers 3 months following the training, they also observed a significant amount of learning in “no-training” observers due to the repeated testing from the three follow-up sessions. It is possible that the first two follow-up sessions (1 week and 1 month after the post-test) helped with the retention of learning in their trained observers. Here, we examined whether additional monthly training facilitates retention of learning. To compare retention with and without additional training, between the post and the retention-test, the five observers in the without-repetition training group also attended three monthly training sessions (i.e., the training sessions took place approximately at 1, 2, and 3 months after the post-test) with the final training session performed on the day before the retention-test.

### Measuring reading speed

We adopted the same sentence pool and testing procedure for measuring reading speed as in previous learning studies (e.g., Yu et al., 2010b,^2017^; Treleaven and Yu, 2020). In each trial, a sentence (average length = 11 words) was randomly selected from a sentence pool without replacement. Using the RSVP method, words of the sentence were presented in a rapid sequence at the same location (left justified) on the screen. Observers read the sentence aloud while maintaining their gaze on a horizontal fixation line. Since the word length ranges from 1 to 14 letters (average = 4), the observers were allowed to move their eyes along the fixation line. Post-stimulus reporting and correction were permitted. Reading accuracy was measured at six exposure durations evenly spaced on a log scale with six sentences per duration. The data were fitted with a Weibull function from which reading speed was calculated based on the exposure duration corresponding to a proportion correct of 0.8.

### Measuring visual-span profile

**FIGURE 2:**
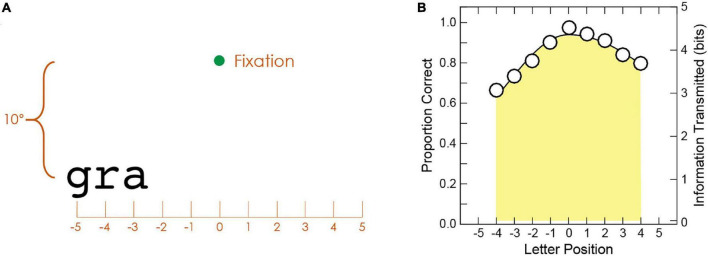
**(A)** Illustration of a trigram trial. Only the fixation point and the trigram were shown on the testing screen. The trigram “gra” is centered at position –4. In this case, three letter responses are collected, one for each of the three letter positions (–5, –4, and –3). **(B)** Sample visual-span profile. The area under the visual-span profile provides the total amount of information transmitted in bits.

We adopted the trigram method (Legge et al., 2007) to acquire visual-span profile (letter recognition accuracy as a function of letter position left or right of midline). The stimuli were trigrams, each composed of three letters randomly selected from 26 English letters without replacement. Given that 99% of the words in the RSVP sentences had nine letters or less, we obtained letter recognition accuracy at nine letter positions using the trigram method, which required presentations of trigrams at 11 positions. As illustrated in Figure 2A, the 11 positions (−5 to 5) were distributed along a horizontal line 10° below fixation. Each trigram was centered at one of the 11 positions and presented for 106 ms. Observers were instructed to report all three letters of the trigram from left to right. From each trigram trial, we were able to gather three responses for the letters situated at three adjacent positions. Twenty trigrams were tested at each position. Only the central nine positions (−4 to 4) accumulated an equal number of letter responses (60 responses). Therefore, only the data collected at these positions were used to construct a visual-span profile. A split Gaussian function was used to fit the data in Figure 2B.

Given the accuracy of letter recognition, the amount of letter information transmitted can be calculated for each letter position, which ranges from 0 bits for chance accuracy (probability of correctly guessing one of 26 letters = 3.8%) to 4.7 bits for 100% accuracy in letter recognition. The conversion formula derived from letter confusion matrices (Beckmann and Legge, 2002), information transmitted in bits = −0.036996 + 4.6761 × accuracy, was used. The area under the visual-span profile (equivalent to summing across the information transmitted by the nine letter positions) was used to quantify the visual span and gave the total amount of information transmitted in bits (Legge et al., 2007). The maximum possible amount of information transmitted through nine letter positions is 41.75 bits.

Using the decomposition analysis (He et al., 2013; Yu et al., 2014), we also examined the three sensory determinants (crowding, mislocations, and resolution) of the size of the visual span. By evaluating the distribution of information loss due to each of these three factors, we can decipher the sensory contributions to the improvement of reading performance.

### Measuring spatial extent of crowding

The spatial extent of crowding was determined by a crowded letter recognition task. The stimuli were trigrams positioned at 10° eccentricity directly below fixation for an exposure duration of 106 ms. We only tested one print size (1°) because the extent of crowding has been shown to be independent of stimulus size (Levi et al., 2002; Tripathy and Cavanagh, 2002). The print size of 1° allowed us to present stimuli at small letter spacing (0.82 × x-width) without significant overlap between adjacent letters. Our data from the isolated letter recognition task confirmed that 1° was above the acuity threshold for all observers (isolated letter recognition accuracy at the corresponding location and letter size = 98%). Accuracy of middle letter recognition was measured at six log-spaced letter spacings (center-to-center distance = 0.82, 1.16, 1.64, 2.32, 3.28, or 4.64 × x-width) with 20 trials per letter spacing. If the observer did not reach 80% accuracy at the largest spacing, additional 20 trials at an even larger spacing (6.56 × x-width) were given at the end. A cumulative-Gaussian function was used to fit the data. The spatial extent of crowding was derived as the center-to-center letter spacing yielding 80% recognition accuracy on the cumulative-Gaussian function.

### Measuring isolated-letter profile

The procedure for measuring the isolated-letter profile was the same as the one for measuring the visual-span profile, except that the stimuli were randomly selected single letters presented at 13 positions (−6 to 6). The data collected at all 13 positions (20 trials/position) were used to construct an isolated-letter profile. Similarly, the size of the isolated-letter span was calculated as the total amount of information transmitted through the isolated-letter profile. The maximum possible amount of information transmitted through 13 letter positions is 60.31 bits.

### Training

Training consisted of five daily sessions (a total of 7,150 trials) of viewing trigrams presented at various letter positions (−5 to 5). Each training session contained 26 blocks. Each block had 55 trials with five trials per letter position. The middle letter of the trigram was designated as the target letter. Before each block, the computer revealed the identity of the target letter through an audio prompt. Within a block, the target letter was always the same, while the left and right letters were selected at random with replacement from trial to trial. As a result, the perceived appearance of the same target letter could vary across trials because of the potentially different interference (crowding) introduced by various combinations of the flanking letters (i.e., the left and right letters). Each of the 26 English letters served as a target letter in one block per session; therefore, there were a total of 26 blocks on each training day.^1^ Observers were instructed to attend to each stimulus and to learn to recognize the target letter.

There was no letter recognition task for observers to perform as they would have already known the identity of the target letter through identity priming. Without examining performance in recognizing random letters, we were unable to track performance change during training. To get some idea about the learning progress during training and also to keep observers more actively engaged, we asked observers to perform a simple stimulus visibility task – providing a subjective rating of target clarity (either “clear” or “not clear”) after each stimulus presentation. We asked each observer to set his/her own criterion for categorizing a trial as “clear” or “not clear” and use it consistently through the training. Observers indicated with a key press if they rated the clarity of the target letter as “not clear.” Otherwise, they continued on to the next trial without any additional key press. There were two training groups: the with-repetition training group and the without-repetition training group. The only distinction between the two training groups was whether the key press triggered a trial replay. In the with-repetition group, each key press was followed by a replay of the stimulus from the preceding trial, and observers were free to repeat the replay as many times as needed through a key press. The without-repetition group did not have the opportunity to review the stimulus. Some letters are more difficult to recognize than others under the same condition (Husk and Yu, 2017). Recognition difficulty also increases for letter positions farther from the midline. Trial replay varies the amount of stimulus exposure to some degree with more exposures for more difficult letters or positions, which possibly leads to an overall larger improvement compared to no trial replay. Observer-reported letter clarity is a subjective measure susceptible to factors like internal criterion and strategy, which can affect how informative the rating is with regard to the observer’s performance during the training. Nevertheless, its variation over the course of learning may still reflect the progress of learning. Following each training block, the percent of “clear” trials (i.e., the percent of trials with target clarity rated as “clear”) along with the current time was displayed on the testing screen.

The average duration taken for a training session (including multiple breaks) was below 45 min for both with-repetition and without-repetition groups. During training, light music was played in the background. Observers’ eye positions were not monitored, resembling the situation of home administration. To motivate them to comply with the training instructions (including maintaining stable fixation at the fixation target), upon the completion of the pre-test, all training observers were informed about an incentive monetary reward based on their post-pre improvements. Good compliance from the observers has also been confirmed through occasional checking of their fixations.

## Results

### Post-pre changes

Before evaluating post-pre changes, we first established that the three observer groups had equivalent baseline (pre-test) performance (Tables 1–4) for all four performance measures (all *p*s > 0.05). As expected, there was a significant effect of print size on RSVP reading speed and isolated-letter-span size (both *p*s < 0.0005). Performance was better at the print size of 2.5° than 1°.

**TABLE 1:** Pre-test performance and performance change (mean ± SEM) in Rapid Serial Visual Presentation (RSVP) reading speed for the control group and the two training groups.

		Pre-test (wpm)	Post/Pre ratio	Retention/Pre ratio
Control	2.5°	182 ± 22	1.17 ± 0.05	/
	1°	79 ± 15	0.98 ± 0.10	/
With-repetition	2.5°	162 ± 21	1.80 ± 0.11	1.51 ± 0.13
	1°	65 ± 12	2.25 ± 0.37	1.80 ± 0.32
Without-repetition	2.5°	166 ± 16	1.77 ± 0.09	1.79 ± 0.24
	1°	70 ± 8	2.14 ± 0.20	2.67 ± 0.28

For the without-repetition group, retention/pre ratio was calculated based on the five observers who returned for the retention-test.

**TABLE 2:** Pre-test performance and performance change (mean ± SEM) in the size of the isolated-letter span for the control group and the two training groups.

		Pre-test (bits)	Post-pre difference	Retention-pre difference
Control	2.5°	59.64 ± 0.16	−0.57 ± 0.26	/
	1°	57.27 ± 0.65	−0.13 ± 0.57	/
With-repetition	2.5°	59.17 ± 0.25	0.60 ± 0.26	0.30 ± 0.20
	1°	57.14 ± 0.46	1.77 ± 0.53	1.27 ± 0.34
Without-repetition	2.5°	59.34 ± 0.32	0.70 ± 0.31	0.65 ± 0.41
	1°	57.67 ± 0.55	1.17 ± 0.62	1.64 ± 0.61

For the without-repetition group, retention/pre difference was calculated based on the five observers who returned for the retention-test.

**TABLE 3:** Pre-test performance and performance change (mean ± SEM) in spatial extent of crowding for the control group and the two training groups.

	Pre-test (× x-width)	Post-pre ratio	Retention/Pre ratio
Control	2.66 ± 0.29	1.09 ± 0.11	/
With-repetition	3.14 ± 0.31	0.71 ± 0.05	0.73 ± 0.03
Without-repetition	2.84 ± 0.38	0.75 ± 0.08	0.69 ± 0.09

For the without-repetition group, retention/pre ratio was calculated based on the five observers who returned for the retention-test.

**TABLE 4:** Pre-test performance and performance change (mean ± SEM) in the size of the visual span for the control group and the two training groups.

	Pre-test (bits)	Post-pre difference	Retention-pre difference
Control	29.17 ± 1.18	2.28 ± 0.44	/
With-repetition	27.52 ± 0.88	7.31 ± 1.01	5.46 ± 1.01
Without-repetition	28.91 ± 0.96	6.69 ± 0.66	7.33 ± 1.01

For the without-repetition group, retention/pre difference was calculated based on the five observers who returned for the retention-test.

**FIGURE 3:**
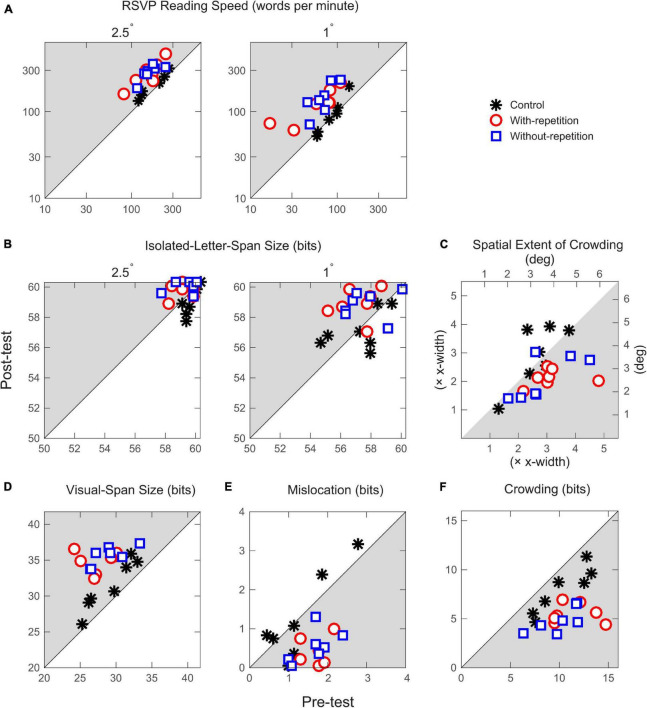
Scatter plots of post-test performance vs. pre-test performance for the three observer groups. **(A)** Rapid Serial Visual Presentation (RSVP) reading speeds at 2.5° and 1° print sizes. Each data point represents one observer. Points on the diagonal line imply no change between the pre- and post-tests. Data points lying inside the shaded region indicate improved performance in the post-test. **(B)** The size of the isolated-letter span at 2.5° and 1° print sizes. The maximum possible amount of information transmitted through 13 letter positions is 60.31 bits. **(C)** Spatial extent of crowding. The top horizontal scale and the right vertical scale show conversion from multiples of x-width to degrees. **(D)** The size of the visual span. The maximum possible amount of information transmitted through nine letter positions is 41.75 bits. **(E)** Mislocation-induced information loss. **(F)**. Crowding-induced information loss.

Figure 3 shows post-test performance as a function of pre-test performance for all four performance measures. The mean post-pre changes for the three groups are listed in Tables 1–4. For RSVP reading speed and spatial extent of crowding, the performance change is expressed as the post-pre ratio. An increase in reading speed from pre-test to post-test is indicated by a ratio larger than 1, whereas improvement for the spatial extent of crowding is expressed by a ratio below 1 (i.e., smaller spatial extent compared to the pre-test). For the visual-span size and the isolated-letter-span size, the performance change is calculated as the post-pre difference.

The post-pre changes for the three observer groups were analyzed using ANOVAs (one-way ANOVAs for the size of the visual span and spatial extent of crowding, and repeated measures ANOVAs for RSVP reading speed and the size of the isolated-letter span). The normality of each group of data was assessed with Shapiro–Wilk Test (Shapiro and Wilk, 1965). According to the Shapiro–Wilk tests, only two groups of data (post-pre ratio in RSVP reading speed at 1° print size and post-pre difference in the size of the visual span) from the with-repetition group appeared to deviate from normality. After identifying the outliers (one for reading speed and two for visual-span size) that caused the non-normality using the median absolute deviation method and threshold recommended by Leys et al. (2013), we repeated the ANOVA analyses, and found that the results did not differ with or without the outliers. Therefore, we included all the data in this report.

As demonstrated in Figure 3, significant group differences were found for all four performance measures (*p*s ≤ 0.008). Bonferroni’s multiple comparison *post-hoc* tests revealed that relative to the control group, both with-repetition and without-repetition training groups showed significant improvements in performance, and there was no difference in post-pre improvements between the two training groups for all four performance measures. For RSVP reading speed and isolated-letter-span size, no significant effect of print size on the post-pre changes was found. The detailed results are described in the following section.

Both training groups exhibited increases in reading speeds compared to the control group (*p*s = 0.0001; Figure 3A). The effect of print size did not reach significance, indicating a complete transfer of learning from the trained print size (2.5°) to the untrained (1°) print size. The improvement was about 80% at the print size of 2.5° and about 120% at 1° (Table 1).

Visual span in both the with-repetition group (*p* < 0.0005) and the without-repetition group (*p* = 0.002) showed significant enlargement relative to the control group, reflected by a larger post-pre difference in the amount of information transmitted in the two training groups (about 7 bits or 1.5 letters; see Figure 3D and Table 4). Using the decomposition analysis, we examined the distribution of information loss due to each of the three sensory determinants (resolution, mislocation, and crowding) of the size of the visual span. At the print size of 2.5°, information loss due to the resolution limit was minimal, as shown by the near-perfect performance in isolated letter recognition (Figure 3B). The majority (91%) of information loss was induced by mislocation and crowding, and crowding alone was responsible for 80%. As shown in Figures 3E,F, both mislocation errors and crowding showed significant reduction following training for the with-repetition group (mislocation: 1.25 ± 0.16 bits, *p* = 0.001; crowding: 5.87 ± 0.92 bits, *p* = 0.005) and the without-repetition group (mislocation: 1.09 ± 0.15 bits, *p* = 0.002; crowding: 5.15 ± 0.56 bits, *p* = 0.026), in comparison to the control group. Apparently, the magnitude of learning benefit is largest for crowding. The two training groups did not differ in post-pre change for any of the sensory determinants.

Besides the reduced information loss, reduction of crowding was also reflected as a smaller spatial extent following training. Both the with-repetition group (a 29 ± 5% reduction in the extent compared to the initial size; *p* = 0.013) and the without-repetition group (25 ± 8%; *p* = 0.031) showed similar significant shrinkage in the spatial extent of crowding in the post-test. To derive the spatial extent, the accuracy of middle letter recognition was obtained at six letter spacings (including the spacing used in training, 1.16 × x-width). We found that performance improvement occurred at untrained spacings as well.

Although the initial isolated-letter-span size was close to the ceiling, we nonetheless found significant enhancement in isolated letter recognition from pre- to post-test for both print sizes in the with-repetition group (*p* = 0.005) and the without-repetition group (*p* = 0.018).

### Training

**FIGURE 4:**
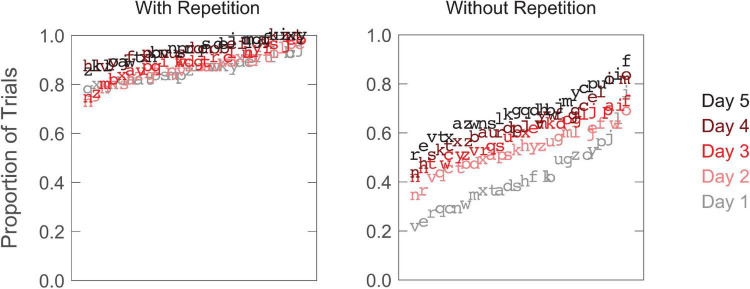
Examples of training data, one observer from each training group. Observer-reported letter clarity (proportion of trials with a clear perception of target letter) is plotted for 26 blocks on each of the 5 training days. Data points are marked by the corresponding target letters, and ordered according to their values, from lowest to highest within each training day.

Without examining observers’ performance in recognizing random letters, we were unable to track performance changes between the pre-test and post-test for the two training groups. However, observers in both groups provided trial-by-trial ratings indicative of the clarity of the target letter, which may reasonably reflect the progress of learning. Figure 4 shows two example data sets, one from each training group. Overall, observers reported that the proportion of trials with a clear perception of the target letter increased with training over the 5 days (with-repetition group: from 0.80 on day one to 0.87 on day five; without-repetition group: from 0.63 to 0.86). The observers in the with-repetition group had the option of replaying a trial as many times as needed. Averaging across letters, the ratio of the total number of replays to the total number of trials for the five training days was 0.48, 0.54, 0.36, 0.32, and 0.28, respectively.

### Retention of learning

Both training groups were evaluated for retention of learning. The only experience difference between the two groups over the 3-month period was that the without-repetition group had three additional monthly training sessions. As indicated in Tables 1–4, when re-tested 3 months following the training, both the with-repetition and the without-repetition training groups showed retention of the improved performance. However, the degree of retention seemed to differ. To evaluate the group difference, we analyzed the retention-post changes for the two training groups using ANOVAs (repeated measures ANOVAs for RSVP reading speed and the size of the isolated-letter span, and one-way ANOVAs for the other two measures). Furthermore, one-tailed, one-sample *t*-tests were employed to assess whether the retention is full or partial. The normality of distribution for each group of data was confirmed with the Shapiro–Wilk normality test.

**FIGURE 5:**
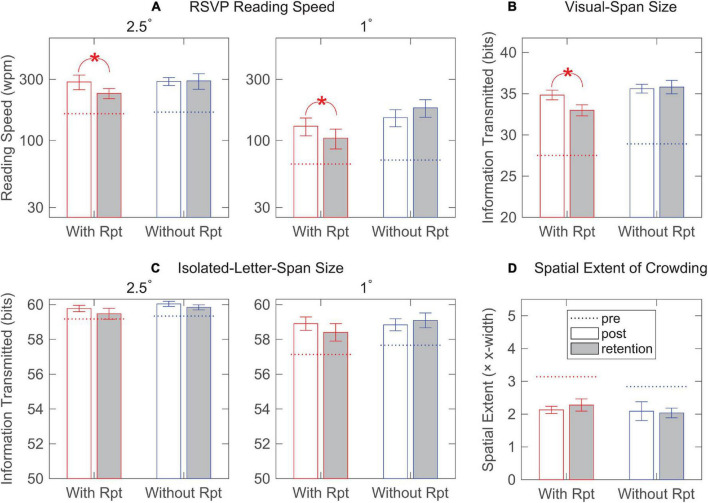
Bar plots showing the group mean of post-test and retention-test performance for both the with-repetition and the without-repetition training groups. **(A)** Rapid Serial Visual Presentation (RSVP) reading speeds at 2.5° and 1° print sizes. The dotted lines represent the group means of pre-test performance. Error bars represent ± SEM. Asterisk (*) indicates a significant difference between post-test and retention-test performance. **(B)** The size of the visual span. **(C)** The size of the isolated-letter span at 2.5° and 1° print sizes. **(D)** Spatial extent of crowding.

As shown in Figure 5, significant group difference was found for RSVP reading speed [*F*(1,10) = 7.94, *p* = 0.02] and visual-span size [*F*(1,10) = 19.16, *p* = 0.001]. One-sample *t*-tests revealed that despite having sizable retention of learning in RSVP reading speed and visual-span size, the with-repetition group lapsed significantly from the post-test to the retention-test (*p*s < 0.0005). No retention-post reduction was found in the spatial extent of crowding and isolated-letter-span size. The without-repetition group, on the other hand, showed full retention of the learning effect in all four measurements. Evidently, the monthly training was sufficient to prevent the deterioration of learning and to enable full retention of performance improvement.

## Discussion

Conventional training task that requires recognizing random letters or words is dispensable for inducing learning in peripheral reading. Stimulating the visual system through repetitive exposure to identity-primed, crowded letters accompanied by a stimulus visibility task enhanced its sensitivity to letter features in the periphery (especially, under crowded conditions), leading to lasting changes in peripheral reading speed of normally sighted young adults. While active engagement was still required from the observers, the visibility task minimized training effort and offered the potential for remote and self-administration. Identity priming, allowing observers to compare each target perception to the true target identity, facilitated learning on a trial-by-trial basis.

Compared to conventional trigram training (e.g., Chung et al., 2004; Yu et al., 2010b), the acquisition of learning in the proposed training was much easier, and remarkably, the efficacy of this new training paradigm was comparable to or greater than that of conventional trigram training. We found a larger improvement in reading speed (near 80% at the trained print size) than in the previous studies (41% in Chung et al., 2004; 54% in Yu et al., 2010b). The total training duration was also shorter (less than 4 h) even though the current training contained more trials (a total of 7,150 trials in the present study vs. 5,200 trials in Chung et al., 2004 and 3,520 trials in Yu et al., 2010b). The with-repetition group on average had 39% more exposure to target letters compared to the without-repetition group. Nevertheless, improvement was similar between the two training groups. Essentially, stimulus replay did not yield a measurable benefit on learning. However, people with central vision loss who often have greater fixation instability than normally sighted people (Culham et al., 1993; Bellmann et al., 2004) may possibly benefit from the with-repetition training, as they can replay stimulus when experiencing a degraded retinal image or missing a stimulus (in whole or part) due to unstable fixation.

### Transfer of learning across print sizes

Learning is more useful if it can be generalized to untrained conditions such as smaller print sizes, especially the ones close to what is encountered in daily life. We found that following our training, learning transferred to the RSVP reading task and also generalized from the trained letter size (2.5°; above the CPS) to untrained letter size (1°) below the CPS. Reading speed at the untrained, smaller print size was slower than the one at the trained, larger print size both before and after the training, indicating the CPS was always larger than 1°. It is possible that CPS stays the same despite the overall improvements in reading speeds (Chung et al., 2004). In any case, it would be desirable for patients who read using their peripheral vision to gain functional reading speed at print sizes smaller than their preferred size. In the present study, all 14 training observers showed 45% or greater improvement at 1° print size. Using 80 wpm as a criterion for functional reading and 160 wpm for fluent reading (Carver, 1992; Rubin, 2013), five of our training observers improved their reading speed from below to above the functional level, and another five improved from functional to fluent reading following the training at the untrained, smaller print size.

### Mechanism of learning

Since our training incorporated both bottom-up (stimulus exposure) and top-down (identity priming or internal feedback) information processing, the learning is possibly associated with the changes that occurred at multiple stages, including the visual processing stage, the decision stage, and connections in between (Shibata et al., 2014). The transfer of learning confirmed that the improvement is certainly not limited to the early encoding stages (Fahle and Poggio, 2002). According to the two-stage model (Shibata et al., 2014), both feature-based plasticity and task-based plasticity may have occurred during the training. The improvements in reading performance may be accounted for by both the refined processing/representation of letter features and the enhancement in the general letter recognition processing.

### The role of crowding in the improved reading performance

As the visual span hypothesis predicted, our data exhibited a strong correlation between the enlargement of visual-span size and the increase of RSVP reading speed (one-tailed Kendall correlation: *r*_τ_ = 0.51; *p* = 0.001 for 2.5° print size; *r*_τ_ = 0.41; *p* = 0.005 for 1° print size). In agreement with previous findings (He et al., 2013; Yu et al., 2014), our results showed that among the three sensory factors, crowding contributes most to the information loss in visual span, and has the largest impact on the enlargement of visual span following the training. To examine the contribution of crowding to the improvement of reading performance, we evaluated the association between the reduction of crowding and the increase in RSVP reading speed amongst all observers. The reduction of crowding can be reflected through a decrease in magnitude (Figure 3F) or a shrinkage in spatial extent (Figure 3C). We found significant, positive correlations (magnitude of crowding: *r*_τ_ = 0.54, *p* = 0.0004 for 2.5° print size and *r*_τ_ = 0.41, *p* = 0.005 for 1° print size; spatial extent of crowding: *r*_τ_ = 0.30, *p* = 0.03 for 2.5° print size and *r*_τ_ = 0.42, *p* = 0.004 for 1° print size), indicating that the reduction in crowding is the key basis of the training-related improvement in reading.

### Subjective rating of target clarity

**FIGURE 6:**
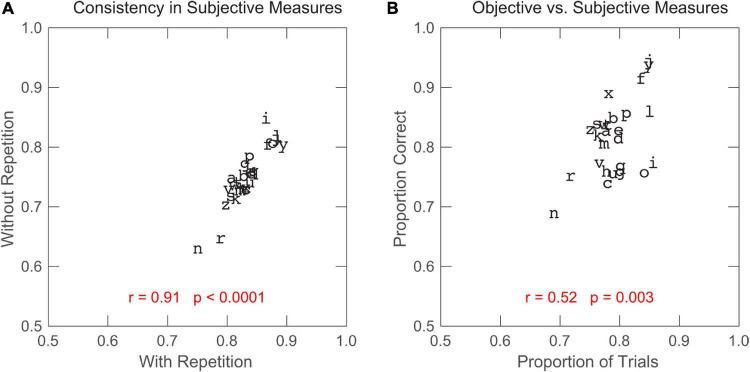
**(A)** Subjective measure (proportion of trials with a clear perception of target letter) for the without-repetition group plotted against the subjective measure for the with-repetition group. **(B)** Objective measure (recognition accuracy of all three letters of trigrams in the post-test) plotted against the subjective measure for the two groups combined. The correlation coefficient and *p*-value (one-tailed) were calculated for each scatter plot.

Subjective rating of target clarity can be considered as a self-evaluation of letter recognition performance. If the subjective rating reflects the observer’s actual performance, we should find a positive correlation between the subjective measure (observers’ subjective rating of target clarity) and the objective, performance-based measure (observers’ letter recognition performance). First, we assessed observer-reported letter clarity from both training groups, and found high agreement between the two groups (one-tailed Pearson correlation: *r* = 0.91, *p* < 0.0001; see Figure 6A). In the subsequent analysis, we compiled the data from all training observers. Indeed, as shown in Figure 6B, a significant, positive correlation exists between the subjective and objective measures (*r* = 0.52, *p* = 0.003). Here, the proportion correct of letter recognition was calculated based on the letter recognition data from the post-test visual-span measurement with all three letters of trigrams being considered. Similar results were also found when we repeated the analysis for recognition of the middle letter of trigram or considered the letter recognition performance from both the pre- and post-tests. Our finding suggests that observer-reported letter clarity can adequately reflect the difficulty of letter recognition. Since observers have a reasonable idea about their actual performance and their need for performance improvement in letter recognition, we can use subjective rating to serve as a guide in training programs, especially those aiming to provide individual customization. For example, we can let observers decide based on their assessment of letter clarity what letters the training should focus on, which may further improve the efficiency of training.

### Retention of learning

Since all observers in the present study have normal central vision, they, unlike patients with central vision loss, have no urge to use their peripheral vision for reading outside the study. This situation gives rise to the possibility that learning may be worn off gradually after the training stops. Even for patients with central vision loss, good retention of learning may still not be guaranteed. The practical value of training would be limited if the learning benefit is only temporary. Consistent with previous training study (Chung et al., 2004), we found that the observers from the with-repetition group were able to retain their learning for at least 3 months with some deterioration in their performance when compared to the post-test. As we discussed, the lapse may be due to the lack of continuous experience in performing a reading-related task using peripheral vision. To examine this speculation, we invited five observers from the without-repetition group to return for four visits, including three monthly training sessions and one retention-test. We found that as a group, the learning was retained at nearly 100%. This finding indicated that monthly training was sufficient to make the learning resilient against the impact of daily reading experience through central vision and to facilitate the full retention of learning.

### Possible further improvement in training efficacy

The efficacy of the current training paradigm may be further promoted by coupling it with other methods or techniques. One potential candidate is the non-invasive brain stimulation approach. A growing body of research has shown that visual perceptual learning can be boosted with concomitant transcranial electric stimulation (Maniglia, 2022). Promising results have been found in both central (Pirulli et al., 2013; Camilleri et al., 2014; Campana et al., 2014; Yang et al., 2022) and peripheral vision (Contemori et al., 2019) in healthy and clinical populations. Another promising method to consider is memory reactivation. Amar-Halpert et al. (2017) found that brief reactivations of consolidated visual memories were sufficient to enable significant learning. After observers encoded and consolidated their memory by participating in the initial full standard practice, a brief training exposure (merely several trials) resulted in learning comparable to what was achieved with full standard practice. Incorporating this procedure may help substantially reduce the number of training trials needed for effective training, which would be especially beneficial for low-vision patients.

## Summary

This study provides the key insight that performing a task of recognizing random letters or words is not a necessity for training peripheral vision to read. The combination of stimulus exposure, identity priming, and visibility task is sufficient to enable learning, complete retention of which is attainable with additional monthly training. The proposed training paradigm simplifies the training task, minimizes task-related frustrations, removes the need on acquiring verbal responses, and reduces training time. In addition, trainees have complete control over the pace of training and only need to press up to three keys to navigate through the whole training. Our novel design, offering simple implementation, capability for remote and self-administration, and an easy translation into low-vision reading rehabilitation, may represent a viable future option for at-home intervention in patients with central vision loss.

## Data availability statement

The data supporting the conclusions of this article are available on request to the corresponding author.

## Ethics statement

The studies involving human participants were reviewed and approved by Institutional Review Board at the Ohio State University. The patients/participants provided their written informed consent to participate in this study.

## Author contributions

DY contributed to the design, data collection and analyses, and manuscript writing and editing.

## Footnotes

1 On training day 2, observer WO7 missed the blocks for letters e, s, t, v, and w and had an extra block for letters c, k, l, r, and z. Results did not differ significantly with or without the inclusion of WO7’s data.
